# A Novel Data-Driven Fault Detection Method Based on Stable Kernel Representation for Dynamic Systems

**DOI:** 10.3390/s23135891

**Published:** 2023-06-25

**Authors:** Qiang Wang, Bo Peng, Pu Xie, Chao Cheng

**Affiliations:** 1 Department of Computer Science and Engineering, Changchun University of Technology, Changchun 130012, China; 2202103061@stu.ccut.edu.cn; 2 Changchun Changguang Yuanchen Microelectronic Technology Co., Ltd., Changchun 130000, China; bo.peng@ycmec.com; 3 Department of Aeronautics and Astronautics, Stanford University, Stanford, CA 94305, USA; xiepu@stanford.edu

**Keywords:** fault detection (FD), distributed framework, sensor networks, data-driven designs, subspace identification, Hellinger distance

## Abstract

With the steady improvement of advanced manufacturing processes and big data technologies, modern industrial systems have become large-scale. To enhance the sensitivity of fault detection (FD) and overcome the drawbacks of the centralized FD framework in dynamic systems, a new data-driven FD method based on Hellinger distance and subspace techniques is proposed for dynamic systems. Specifically, the proposed approach uses only system input/output data collected via sensor networks, and the distributed residual signals can be generated directly through the stable kernel representation of the process. Based on this, each sensor node can obtain the identical residual signal and test statistic through the average consensus algorithms. In addition, this paper integrates the Hellinger distance into the residual signal analysis for improving the FD performance. Finally, the effectiveness and accuracy of the proposed method have been verified in a real multiphase flow facility.

## 1. Introduction

As a result of intelligence and informatization, modern industrial processes have evolved to become more complicated. Any abnormal behavior of equipment components may affect productivity or even cause accidents. To guarantee the reliability and stability of industrial systems, fault detection (FD) plays a fundamental role and has received intensive attention from scholars and engineers [1,2,3].

Currently, the majority of FD methods are commonly classified as model-based techniques and data-driven techniques [4]. In the framework of model-based FD, it is necessary to obtain a precise mathematical representation of the systems. In addition, the accuracy of the FD results will depend on the accuracy of the modeling. According to the way of signal generation, existing model-based FD methods can be divided into three distinct groups, i.e., parameter estimation techniques, observer-based techniques, the subspace-based strategy [5]. In practical applications, such methods can successfully implement FD schemes when accurate mathematical models are available. Unfortunately, with the increasing size of modern engineering systems, the modeling of systems using first principles poses further challenges.

With the accelerated advancement of sensor networks and data processing methods, data-driven FD strategies have naturally become a critical research topic and are well developed [6,7,8,9,10]. Data-driven FD techniques are typically sorted into multivariate statistics, neural networks, and subspace-technique-aided schemes, etc. [11]. Specifically, traditional multivariate statistical methods [12,13,14], i.e., canonical correlation analysis (CCA), have been extensively studied and applied to modern industrial systems. The core of multivariate statistical methods is to analyze the correlation among process variables, followed by constructing appropriate test statistics for FD tasks. This group of methods can solve the FD problem well in static processes. However, such traditional FD strategies do not usually consider dynamic changes in the systems. Therefore, they are usually unable to perform FD tasks in dynamic systems. In the past few years, with increasing attention to statistical learning, neural network-based FD methods have been rapidly developed [15,16,17,18]. This group of methods usually uses historical data generated by the production process to train the neural network model. Then, the models classify the test data to determine whether faults have occurred. Due to the excellent fitting ability of neural networks, neural network-based methods have superior performance in dealing with FD problems for nonlinear systems. However, the training phase of neural networks requires the use of abundant labeled data, which has some limitations in practical applications [19]. In recent years, the subspace-technique-aided FD method has been widely studied because of its simple design and lower computational effort [20,21,22,23,24,25]. The core idea behind it is to identify the system parameters through the collected data. Because it takes into account the dynamic behavior in the systems, the subspace-aided FD method performs very well in dealing with process dynamics. In the subspace technique framework, Reference [23] proposes a stable kernel representation (SKR) of the systems. Remarkably, the proposed SKR scheme can directly construct residual generators using process data without identifying complex system models. Based on this, many data-driven FD algorithms are designed under the SKR framework.

At present, the majority of data-driven FD methods have been designed in a centralized framework, which involves collecting all process data in a central location to carry out necessary computations and FD tasks. With the growth of industrial process size, the centralized design becomes increasingly demanding in terms of memory and computing power, resulting in poor flexibility and high cost. Therefore, there is a strong research increase in distributed data-driven FD [26]. For example, ref. [27] evaluated the effectiveness of multiblock multivariate statistics-based approaches, such as PCA and PLS, for decentralized monitoring and assessed the individual contributions of each block. Although the above approaches can implement distributed FD for each sub-block, they do not take into account the connection among the sub-blocks. On this basis, ref. [28] designed a multiblock PCA strategy where information interaction among subblocks is considered. Similarly, considering the connection among neighboring nodes, ref. [29] designed a distributed CCA algorithm to achieve plant-wide process monitoring. The core idea behind it was to reduce uncertainty through information interaction among neighboring nodes. However, when there are few relevant variables in the historical data, the distributed FD results obtained based on this method are usually unreliable. In order to address this defect, ref. [30] designed a distributed, regularized CCA-based process monitoring algorithm. First, the traditional CCA algorithm is executed between the local and the neighboring nodes. In order to eliminate uncorrelated variables from the monitoring data, a GA-based regularization algorithm is then embedded in the traditional CCA technique. Finally, according to the local monitoring results, the corresponding residual signals and test statistics can be generated at the local node. In terms of technical systems, the multiphase flow facility used in this study is a device to achieve the separation of water, oil, and air at a given flow rate. The test zone, consisting of splitters and supply lines, can provide a mixture of water, oil, and air. The technical system is typically used to verify the effectiveness and accuracy of process monitoring and FD algorithms, such as the latent-variable-analysis-based FD method [31], the Kalman-filter-based FD method [32], and the multivariate-statistics-based process monitoring method [33]. In addition, the technical system used is described in detail in [34,35].

In general, the data-driven distributed FD methods above usually ignore the dynamic changes in the process. Therefore, these data-driven distributed methods have some limitations in dealing with FD problems in dynamic processes. In addition, there are abundant process data in dynamic systems, and the relationship among process variables is complicated. The abundant process data and strong coupling among variables bring new challenges to existing distributed data-driven FD solutions.

Motivated by the aforementioned points, a new data-driven distributed FD strategy is developed for dynamic systems. Compared with previous research, the key contributions of the developed FD solution are given as

1. Compared with traditional SKR-based FD approaches, the proposed method is more sensitive to fault information by introducing the Hellinger distance (HD) in the residual signal.
2. The consensus algorithm is embedded in the information interaction among sensor blocks. Therefore, each sensor block can obtain FD results without global fusion operations, thus remarkably improving FD efficiency.
3. It has superior flexibility in the design of FD framework, particularly when the system models are not accurately obtained.

The structuring of this work is structured as follows. Section 2 provides information about the system descriptions, Hellinger distance, and the average consensus algorithms. In Section 3, a new distributed FD scheme for dynamic systems is presented. The effectiveness of the proposed FD algorithm is then demonstrated through a multiphase flow facility in Section 4. Finally, Section 5 presents the conclusion and prospects for future work.

## 2. Preliminaries

### 2.1. System Descriptions

Given a LTI system H(z) with input factor $u\in R^{u}$ and output factor $y\in R^{y}$, the input–output (I/O) behavior is characterized as

(1) $$\begin{array}{c} y(z)=H(z)u(z) \end{array}$$

where variable *z* represents the complex z-transform. In order to analyze the relationship among variables in dynamic systems, the state space model is used in this study. It not only reflects dynamic behavior in the process data but also provides a concise description, which is usually expressed in a standard form, as follows:

(2) $$\begin{array}{c} x\left(k+1\right)=\left[\begin{array}{cc} \;A & B\; \end{array}\right]\left[\begin{array}{c} x(k)\\ u(k) \end{array}\right] \end{array}$$

(3) $$\begin{array}{c} y\left(k\right)=\left[\begin{array}{cc} \;C & D\; \end{array}\right]\left[\begin{array}{c} x(k)\\ u(k) \end{array}\right] \end{array}$$

where *A*, *B*, *C* and *D* are system parameters; $u\left(k\right)\in R^{u}$, $y\left(k\right)\in R^{y}$, and $x\left(k\right)\in R^{x}$ refer to the system input, output, and state variables, respectively.

**Figure 1:**
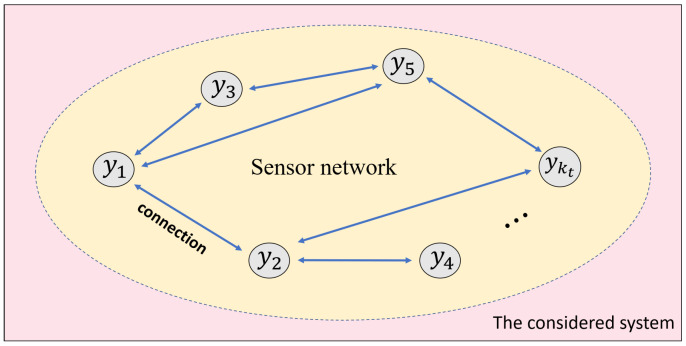
A dynamic system equipped with a sensor network.

A sensor network consisting of $k_{t}$ blocks has been integrated into the considered system, as shown in Figure 1. In the sensor network, the network topology G can be represented using “node” J and “edge” K as

(4) $$\begin{array}{c} G=(J,K) \end{array}$$

where J$=\{1,\cdots ,k_{t}\}$ depicts the set of sensor blocks; K∈{δ×δ} depicts the set of edges.

In order to obtain the sef of adjacent blocks, J in (1) is further sketched as

(5) $$\begin{array}{c} J_{i}=\left\{i\in J|\left(i,j\right)\in K\right\} \end{array}$$

where $J_{i}$ depicts all adjacent blocks of the *i*-th block.

### 2.2. Hellinger Distance

Hellinger distance (HD), also known as the Bhattacharyya distance, is a type of *f*-divergence [36]. The *f*-divergence is a function that measures the difference between two probability distributions. HD is a statistical technique that evaluates the resemblance of two frequency distributions to each other. Supposing that n(x) and m(x) denote two probability density functions (PDFs) and since the probability distributions of the variables are unknown in the general definition, the HD between n(x) and m(x) is next defined as

(6) $$\begin{array}{c} H\left(n,m\right)=\sqrt{\frac{1}{2}\int_{-\infty }^{+\infty }{\left(\sqrt{n(x)}-\sqrt{m(x)}\right)}^{2}dx} \end{array}$$

which can be also be expressed in Euclidean norm

(7) $$\begin{array}{c} H\left(n,m\right)=\frac{1}{\sqrt{2}}\|\sqrt{n}-\sqrt{m}{\|}_{2} \end{array}$$

Based on the Cauchy inequality, HD is a symmetric bounded metric which satisfies $0\leq H\left(n,m\right)\leq 1$ and H(n,m)=H(m,n). In addition, according to the Lebesgue theorem, the squared form of HD in (6) is characterized as

(8) $$\begin{array}{c} H^{2}\left(n,m\right)=1-\int_{-\infty }^{+\infty }\sqrt{n(x)m(x)}dx \end{array}$$

In order to perform FD task, Lemma 1 gives a concise representation of (8), which serves as the basis of the proposed approach.

**Lemma** **1.** *Consider two PDFs $n\left(x\right)∼N\left(\mu_{n},{\sigma^{2}}_{n}\right)$ and $m\left(x\right)∼N\left(\mu_{m},{\sigma^{2}}_{m}\right)$, $H^{2}\left(n,m\right)$ is further represented as follows:*

(9) $$\begin{array}{c} H^{2}\left(n,m\right)=1-\left(\frac{2\sigma_{n}\sigma_{m}}{{\sigma_{n}}^{2}+{\sigma_{m}}^{2}}\right)\exp\left(-\frac{{\left(\mu_{n}-\mu_{m}\right)}^{2}}{4({\sigma_{n}}^{2}+{\sigma_{m}}^{2})}\right) \end{array}$$

### 2.3. Average Consensus Algorithm

Given a communication network consisting of $k_{t}$ nodes, the consensus algorithm is a convergence technique to implement consensus calculations. For a data vector $\mu_{i}$ at the *i*-th sensor block, the consensus technique can be executed as

(10) $$\begin{array}{c} \mu_{i}\left(s+1\right)=v_{i,i}\mu_{i}\left(s\right)+\sum_{j\in J_{i}}v_{i,j}\mu_{j}\left(s\right),i=1,\cdots ,k_{t} \end{array}$$

where $\mu_{i}\left(s\right)$ refers to the calculated value of $\mu_{i}$ during the *s*th iteration; $v_{i,j}$ is the weighting coefficients,. Many studies [37,38,39] have designed algorithms to solve the weighting problem. Among them, the Metropolis–Hastings technique not only speeds up the convergence of the iterative algorithm but also enables computation in a distributed manner. Therefore, the Metropolis–Hastings technique is used to construct the weighting factors in this paper. The weighting factors are assembled as follows

(11) $$v_{ij}=\left\{\begin{array}{cc} \frac{1}{\max\{j_{i},j_{j}\}+1} & j\in J_{i}\\ 0 & \;\;\;j\notin J_{i}\;\;and\;\;j\neq i\\ 1-\sum_{k\neq i}v_{ik} & i=j \end{array}\right.$$

where $J_{i}$ represents all adjacent blocks of the *i*-th block. $j_{i}=\left|J_{i}\right|$ as the cardinality of $J_{i}$. Let *V* be

(12) $$\begin{array}{c} \left[\begin{array}{ccc} v_{1,1} & \cdots & v_{1,k_{t}}\\ \vdots & \ddots & \vdots \\ v_{k_{t},1} & \cdots & v_{k_{t},k_{t}} \end{array}\right]\in R^{k_{t}\times k_{t}}. \end{array}$$

The final consensus results can be presented as

(13) $$\begin{array}{c} \lim_{s\to \infty }\left[\begin{array}{c} \mu_{1}\left(s\right)\\ \vdots \\ \mu_{k_{t}}\left(s\right) \end{array}\right]=1⊗\left(\frac{1}{k_{t}}\sum_{i=1}^{k_{t}}\mu_{i}\left(0\right)\right) \end{array}$$

which indicates that the consensus value of each block will converge to the average value of all sensor blocks.

## 3. Methodology

In this section, the SKR of the residual generator is first introduced. Considering the probability distribution of fault information, a novel FD strategy is then presented and applied to the FD problem.

### 3.1. SKR

Considering the process model of (1)–(3) above, the left coprime factorizations (LCF) of H(z) is given as follows:

(14) $$\begin{array}{c} H\left(z\right)=D+C{\left(zI-A\right)}^{-1}B=P^{-1}\left(z\right)Q\left(z\right) \end{array}$$

where $\left(P(z),Q(z)\right)$ is called the left coprime pair. A key feature of the LCF under noise-free and fault-free conditions is displayed as

(15) $$\begin{array}{c} \left[\begin{array}{cc} -P(z) & Q(z) \end{array}\right]\left[\begin{array}{c} u(z)\\ y(z) \end{array}\right]=0 \end{array}$$

where $\left[\begin{array}{cc} -P(z) & Q(z) \end{array}\right]$ is denoted as the SKR of (2) and (3) [23]. Therefore, all LTI residual generators can be parameterized as

(16) $$\begin{array}{c} r\left(z\right)=\left[\begin{array}{cc} -P(z) & Q(z) \end{array}\right]\left[\begin{array}{c} u(z)\\ y(z) \end{array}\right]=0 \end{array}$$

### 3.2. Data-Driven Distributed Fault Detection

Given that a sensor network is integrated into the dynamic system, the state-space model of the system and sensor measurements with noise are displayed as

(17) $$\begin{array}{c} x(k+1)=Ax(k)+Bu(k)+\sigma (k) \end{array}$$

(18) $$\begin{array}{c} y_{i}\left(k\right)=\left\{\begin{array}{c} L_{i}x\left(k\right)+\tau_{i}\left(k\right),\;\mathrm{fault}-\mathrm{free}\\ L_{i}\left(x(k)+f(k)\right)+\tau_{i}\left(k\right),\;\mathrm{faulty} \end{array}\right. \end{array}$$

where $x\left(k\right)\in R^{k_{l}}$, $u\left(k\right)\in R^{k_{m}}$ denote the system state and the process input; $y_{i}\left(k\right)\in R^{k_{n}}$ is the output vector at the *i*-th sensor sub-block; $f\left(k\right)\in R^{k_{f}}$ denotes the unknown faults; $\sigma \left(k\right)∼\;N\left(0,\Sigma_{\sigma }\right)$ and $\tau_{i}\left(k\right)∼\;N\left(0,\Sigma_{\tau_{i}}\right)$ represent process and measurement noise, respectively. In addition, σ(k) and $\tau_{i}\left(k\right)$ are assumed to be Gaussian distributions.

Considering that measurement data $y_{i}$, $i=1,\cdots k_{t}$, can be sent to a sensor block, a global model is then constructed as follows:

(19) $$\begin{array}{c} y\left(k\right)=\left\{\begin{array}{c} Lx\left(k\right)+\tau \left(k\right),\;\mathrm{fault}-\mathrm{free}\\ L\left(x(k)+f(k)\right)+\tau \left(k\right),\;\mathrm{faulty} \end{array}\right. \end{array}$$

where

$$y\left(k\right)=\left[\begin{array}{c} y_{1}\left(k\right)\\ \vdots \\ y_{k_{t}}\left(k\right) \end{array}\right],\;L=\left[\begin{array}{c} L_{1}\\ \vdots \\ L_{k_{t}} \end{array}\right],\;\tau \left(k\right)=\left[\begin{array}{c} \tau_{1}\left(k\right)\\ \vdots \\ \tau_{k_{t}}\left(k\right) \end{array}\right].$$

To complete the algorithm implementation of SKR, data models are indispensable in the design processes [40]. Assuming there exists a data variable $\kappa_{s}\left(k\right)\in R^{k_{\kappa }}$, it can be further depicted as

(20) $$\begin{array}{c} \kappa_{s}\left(k\right)={\left[\begin{array}{ccc} \kappa^{T}\left(k\right) & \cdots & \kappa^{T}\left(k+s\right) \end{array}\right]}^{T}\in R^{\left(s+1\right)k_{\kappa }} \end{array}$$

(21) $$\begin{array}{c} \Upsilon_{k}={\left[\begin{array}{ccc} \kappa (k) & \cdots & \kappa (k+N-1) \end{array}\right]}^{T}\in R^{k_{\kappa }\times N} \end{array}$$

(22) $$\begin{array}{c} \Upsilon_{k,s}={\left[\begin{array}{ccc} \kappa_{s}\left(k\right) & \cdots & \kappa_{s}\left(k+N-1\right) \end{array}\right]}^{T}\in R^{\left(s+1\right)k_{\kappa }\times N} \end{array}$$

where *k* denotes sampling instants, and *s* and *N* are some integers.

According to the extended models of H(z) in (17) and (18), a data model is derived by iterative computation at each node:

(23) $$\begin{array}{c} Y_{s,i}=F_{s,i}X_{k}+G_{s,i}U_{k,s}+H_{s,i}\Theta_{k,s}+\Xi_{s,i}\in R^{\left(s+1\right)k_{n}\times N} \end{array}$$

where

$$F_{s,i}=\left[\begin{array}{c} L_{i}\\ L_{i}A\\ \vdots \\ L_{i}A^{s} \end{array}\right],$$

$$G_{s,i}=\left[\begin{array}{cccc} 0 & 0 & \cdots & 0\\ L_{i}B & \ddots & \ddots & \vdots \\ \vdots & \ddots & \ddots & 0\\ L_{i}A^{s-1}B & \cdots & L_{i}B & 0 \end{array}\right],$$

$$\Xi_{s,i}=\left[\begin{array}{ccc} \tau_{i}\left(k\right) & \cdots & \tau_{i}\left(k+N-1\right)\\ \vdots & \ddots & \vdots \\ \tau_{i}\left(k+s\right) & \cdots & \tau_{i}\left(k+s+N-1\right) \end{array}\right].$$

In order to remove the unobservable variable $X_{k}$, (23) is re-modeled as

(24) $$\begin{array}{c} \left[\begin{array}{c} U_{k,s}\\ Y_{s,i} \end{array}\right]=K_{s,i}\left[\begin{array}{c} U_{k,s}\\ X_{k} \end{array}\right]+\left[\begin{array}{c} 0\\ H_{s,i}\Theta_{k,s}+\Xi_{s,i} \end{array}\right] \end{array}$$

where $K_{s,i}=\left[\begin{array}{cc} I & 0\\ G_{s,i} & F_{s,i} \end{array}\right]\in R^{\left(s+1\right)\left(k_{n}+k_{m}\right)\times \left(n+\left(s+1\right)k_{m}\right)}$. When *s* is large enough, there must be a left nullspace of $K_{s,i}$ as

$${K_{s,i}}^{⊥}K_{s,i}=0,{K_{s,i}}^{⊥}\in R^{\left(\left(s+1\right)k_{n}-k_{l}\right)\times \left(s+1\right)\left(k_{n}+k_{m}\right)}\Rightarrow$$

(25) $$\begin{array}{c} {K_{s,i}}^{⊥}\left[\begin{array}{c} U_{k,s}\\ Y_{s,i} \end{array}\right]=\left[\begin{array}{cc} {K_{s,i,u}}^{⊥} & {K_{s,i,y}}^{⊥} \end{array}\right]\left[\begin{array}{c} U_{k,s}\\ Y_{s,i} \end{array}\right]=\left[\begin{array}{c} 0\\ {K_{s,i}}^{⊥}\left(H_{s,i}\Theta_{k,s}+\Xi_{s,i}\right) \end{array}\right]. \end{array}$$

${K_{s,i}}^{⊥}$ is called the SKR of the system. Due to the excellent reliability and robustness of QR algorithm, the data-driven implementation of ${K_{s,i}}^{⊥}$ is able to be executed via QR decomposition and SVD:

(26) $$\begin{array}{c} \left[\begin{array}{c} T_{k-s_{p},s_{p}-1,i}\\ U_{k,s}\\ Y_{s,i} \end{array}\right]=\left[\begin{array}{ccc} R_{1,1} & 0 & 0\\ R_{2,1} & R_{2,2} & 0\\ R_{3,1} & R_{3,2} & R_{3,3} \end{array}\right]\left[\begin{array}{c} Q_{1,1}\\ Q_{2,1}\\ Q_{3,1} \end{array}\right] \end{array}$$

(27) $$\begin{array}{c} \left[\begin{array}{cc} R_{2,1} & R_{2,2}\\ R_{3,1} & R_{3,2} \end{array}\right]=\left[\begin{array}{cc} V_{1} & V_{2} \end{array}\right]\left[\begin{array}{cc} \Psi_{1,1} & 0\\ 0 & \Psi_{2,2} \end{array}\right]\left[\begin{array}{c} {D_{1}}^{T}\\ {D_{2}}^{T} \end{array}\right] \end{array}$$

where $s_{p}$ represents the past moment; $T_{k-s_{p},s_{p}-1,i}=\left[\begin{array}{c} U_{k-s_{p},s_{p}-1}\\ Y_{k-s_{p},s_{p}-1,i} \end{array}\right]$ is an I/O dataset of the past $s_{p}$ moments.

In addition, the noise terms can be identified by the proof in [20]:

(28) $$\begin{array}{c} R_{3,3}Q_{3,1}=H_{s,i}\Theta_{k,s}+\Xi_{s,i} \end{array}$$

Observe that $\left[\begin{array}{cc} R_{2,1} & R_{2,2}\\ R_{3,1} & R_{3,2} \end{array}\right]$ and $K_{s,i}$ have same null space. It thus holds that

(29) $$\begin{array}{c} \Psi_{2,2}\approx 0,\;\;{K_{s,i}}^{⊥}={V_{2}}^{T}\in R^{\left(\left(s+1\right)k_{n}-k_{l}\right)\times \left(s+1\right)\left(k_{n}+km\right)}. \end{array}$$

In order to identify the residual signal, ${K_{s,i,y}}^{⊥}$ in (25) needs to be obtained in a data-driven manner. It has been demonstrated [40] that

(30) $$\begin{array}{c} {K_{s,i,y}}^{⊥}F_{s,i}=0,\;{K_{s,i,u}}^{⊥}=-{K_{s,i,y}}^{⊥}G_{s,i} \end{array}$$

where ${K_{s,i,y}}^{⊥}={F_{s,i}}^{⊥}$. Therefore, the residual generator can be obtained as follows:

(31) $$\begin{array}{c} {K_{s,i,y}}^{⊥}\left(H_{s,i}\Theta_{k,s}+\Xi_{s,i}\right)={K_{s,i,y}}^{⊥}R_{3,3}Q_{3,1} \end{array}$$

(32) $$\begin{array}{c} r_{i}\left(k\right)={K_{s,i,y}}^{⊥}y_{s}\left(k\right)-{K_{s,i,u}}^{⊥}u_{s}\left(k\right). \end{array}$$

Although the residual signal $r_{i}\left(k\right)$ generated by SKR has the advantages of having a simple design and a low computational effort, the robustness of its FD results often becomes weak under actual operating conditions. In order to improve the robustness of the SKR framework for FD applications, the probability distribution of the residual signal deserves further investigation. Based on the idea of HD, an approach to evaluate the similarity between two PDFs is introduced into the SKR framework.

Considering that the above process noise and measurement noise obey Gaussian distributions, the residual signal is $r_{i}∼\left(0,{\Lambda_{i}}^{2}\right)$ in the normal (fault-free) historical dataset. In addition, for the actual fault dataset, the residual signal is ${\hat{r}}_{i}∼\left({\hat{\vartheta }}_{i},{{\hat{\Lambda }}_{i}}^{2}\right)$.

Therefore, a HD metric for the reisdual signal $r_{i}$ at the *i*-th sensor block can be represented as follows:

(33) $$\begin{array}{c} {h_{i}}^{2}=1-\int_{-\infty }^{+\infty }\sqrt{f\left(r_{i}\right)f\left({\hat{r}}_{i}\right)dr_{i}} \end{array}$$

where $f(r_{i})$ and $f({\hat{r}}_{i})$ denote the PDFs of $r_{i}$ and ${\hat{r}}_{i}$, respectively.

According to the property of Lemma 1, (33) is able to be rewritten as

(34) $$\begin{array}{c} {h_{i}}^{2}=1-{\left(\frac{2\Lambda_{i}{\hat{\Lambda }}_{i}}{{\Lambda_{i}}^{2}+{{\hat{\Lambda }}_{i}}^{2}}\right)}^{1/2}\exp\left(-\frac{{\left(\vartheta_{i}-{\hat{\vartheta }}_{i}\right)}^{2}}{4({\Lambda_{i}}^{2}+{{\hat{\Lambda }}_{i}}^{2})}\right) \end{array}$$

Based on the proposed FD algorithm, the $T^{2}$ statistic at each sensor block can be displayed as

(35) $$\begin{array}{c} {T_{i}}^{2}=\left({{\hat{h}}_{i}}^{2}-\tilde{{h_{i}}^{2}}\right)\Psi^{-1}{\left({{\hat{h}}_{i}}^{2}-\tilde{{h_{i}}^{2}}\right)}^{T} \end{array}$$

where ${{\hat{h}}_{i}}^{2}\in R^{k_{n}}$ can be obtained by (34) under the actual fault dataset; $\tilde{{h_{i}}^{2}}\in R^{k_{n}}$ denotes the mean term of ${h_{i}}^{2}$ under the normal historical dataset; Ψ is the covariance matrix of ${{\hat{h}}_{i}}^{2}-\tilde{{h_{i}}^{2}}$.

In order to implement distributed FD, each sensor node needs to perform the identical $T^{2}$ test statistic. Based on the above purpose, the average consensus technique is introduced in this framework. The consensus algorithm for $\varpi_{i}={{\hat{h}}_{i}}^{2}-\tilde{{h_{i}}^{2}}$ is

(36) $$\begin{array}{c} \varpi_{i}\left(s+1\right)=v_{i,i}\varpi_{i}\left(s\right)+\sum_{j\in J_{i}}v_{i,j}\varpi_{j}\left(s\right),i=1,\cdots ,k_{t} \end{array}$$

where *s* denotes the iteration number. The initial value is $\varpi_{i}\left(0\right)=\varpi_{i}$. Furthermore, it holds that [5]

(37) $$\begin{array}{c} \lim_{s\to \infty }\varpi_{i}\left(s+1\right)=\bar{\varpi }=\frac{1}{k_{t}}\sum_{i=1}^{k_{t}}\varpi_{i}=\frac{1}{k_{t}}\sum_{i=1}^{k_{t}}\left({{\hat{h}}_{i}}^{2}-\tilde{{h_{i}}^{2}}\right) \end{array}$$

As the algorithm runs until convergence, the consensus result can be obtained at each block:

(38) $$\begin{array}{c} \varpi_{i}=\frac{1}{k_{n}}\sum_{i=1}^{k_{n}}({{\hat{h}}_{i}}^{2}-\tilde{{h_{i}}^{2}}) \end{array}$$

Based on the consensus techniques above, identical $\varpi_{i}$ is obtained at each node. Therefore, (35) can be rewritten as

(39) $$\begin{array}{c} {T_{i}}^{2}=\varpi_{i}\phi^{-1}{\varpi_{i}}^{T} \end{array}$$

where ϕ denotes the the covariance matrix of $\varpi_{i}$.

As a result, the $T^{2}$ statistic can be executed in parallel at each block. When the amount of data is sufficient, the used $T^{2}$ statistic obeys a chi-square distribution ($T^{2}∼{\chi_{\beta }}^{2}\left(k_{n}\right)$). Specifically, $\chi_{\beta }$ can be determined by a $\chi^{2}$ distribution with degrees $k_{n}$ of freedom as follows:

(40) $$\begin{array}{c} prob\left\{\chi >\chi_{\beta }\right\}=\beta \Leftrightarrow prob\left\{\chi \leq \chi_{\beta }\right\}=1-\beta \end{array}$$

Based on this, the fault detection threshold can be calculated as

(41) $$\begin{array}{c} J_{th}={\chi_{\beta }}^{2}\left(k_{n}\right) \end{array}$$

where $k_{n}$ is the dimension of the residual data; and β is the confidence level (acceptable false alarm rate).

In addition, the FD logic for each node is represented as follows:

(42) $$\begin{array}{c} \left\{\begin{array}{c} {T_{i}}^{2}<J_{th}\Rightarrow \mathrm{fault}-\mathrm{free},\\ {T_{i}}^{2}\geq J_{th}\Rightarrow \mathrm{faulty}. \end{array}\right. \end{array}$$

**Figure 2:**
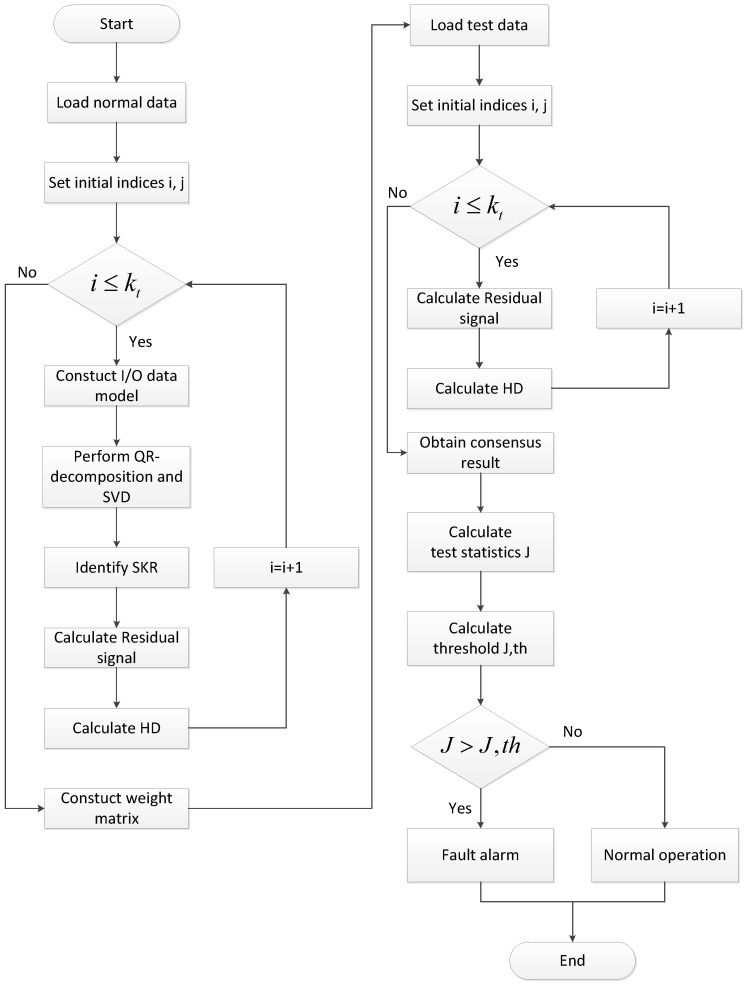
The flow chart of the proposed fault detection algorithm.

In summary, with the help of the SKR framework, Hellinger distance, and average consensus algorithm, the distributed FD scheme is summarized in Algorithms 1 and 2. In addition, the flow chart of the proposed FD algorithm is shown in Figure 2.

**Algorithm 1:** Off-Line Phase.
S1. Load the normal (fault-free) data.;
S2: Set two indices i,j where $i=1,\cdots ,k_{t}$ and $j\in J_{i}\cup {\bar{J}}_{i}$;
S3: **while** $i\leq k_{t}$ **do**
S4: Constuct I/O data model at each node via (23);
S5: Perform QR-decomposition (26) and SVD (27);
S6: Identify SKR ${K_{s,i}}^{⊥}$ via (29) and (30);
S7: Obtain the residual signals $r_{i}$ at each sensor node;
S8: Calculate Hellinger distance ${h_{i}}^{2}$ for each residual signal via (34);
S9: **end while**
S10: Constuct weight matrix *V* via (11).


**Algorithm 2:** Online Phase.
S1. Load the actual test data.;
S2: **while** $i\leq k_{t}$ **do**
S3: Obtain the residual signals ${\hat{r}}_{i}$ under the acutal fault case;
S4: Calculate Hellinger distance ${{\hat{h}}_{i}}^{2}$ for each residual signal via (34);
S5: **end while**
S6: Calculate $\bar{\varpi }=\frac{1}{k_{t}}\sum_{i=1}^{k_{t}}\tilde{({{\hat{h}}_{i}}^{2}-{h_{i}}^{2}})$ using the average consensus algorithm (36);
S7: Obtain the identical $T^{2}$ test statistic via (39);
S8: Make a FD logic decision whether a fault has occurred based on (41).


## 4. Case Study

### 4.1. Facility Description

**Figure 3:**
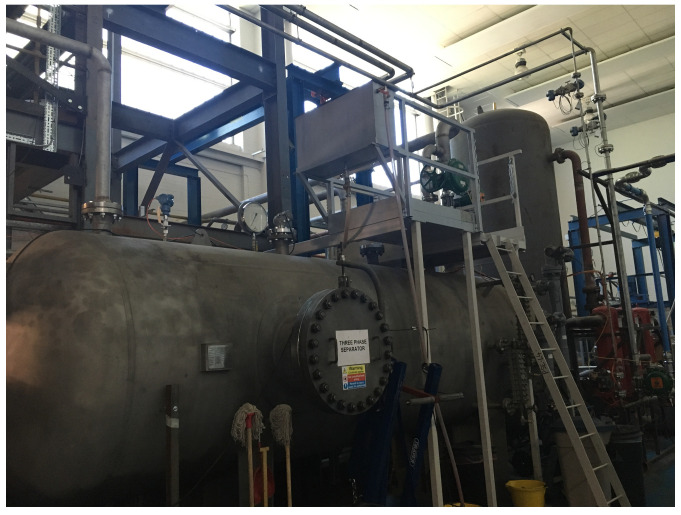
An overview of the multi-phase flow facility.

**Figure 4:**
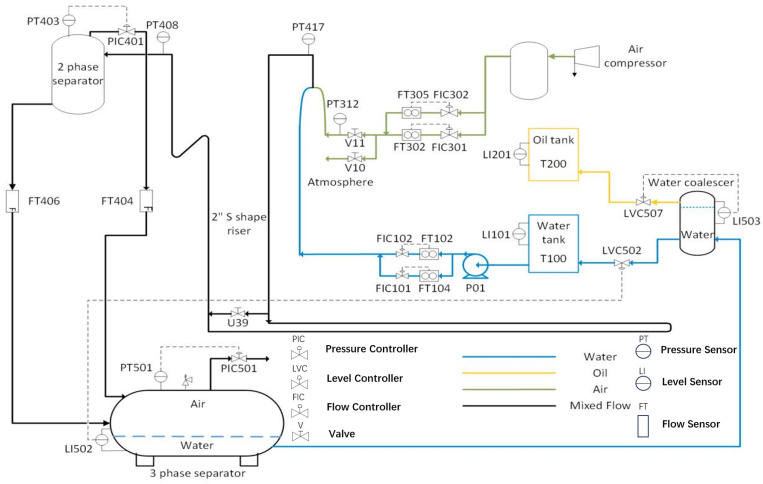
A schematic of the multiphase flow facility [35] (see Table 1 for tag descriptions).

In this study, a data set obtained from a multi-phase flow plant [35] is used to validate the proposed FD algorithm. The multiphase flow plant can achieve gas–liquid separation at a given flow rate. This device takes into account various working conditions during operation, so it can generate abundant process data from different operating conditions. In addition, the generated process data contain dynamic behavior by changing the set point of the flow rate. It is depicted in Figure 3, and its schematic diagram is presented in Figure 4. Specifically, the device comprises geometrically designed pipes and a 1.2 m high liquid–gas splitter. It is capable of providing separate air, oil, and water, as well as mixtures of these fluids. During the operation of the plant, the mixtures are split in a horizontal splitter. The air is returned to the environment, while the water–oil mixture is returned to their respective tanks (T100 and T200). The water coalescers ensure complete separation of oil and water before returning to their respective tanks. The flow conditions of air, water, and oil can be regulated by control valves. In addition, the relevant control valves can be operated continuously between closing and opening. In terms of sensor distribution, there are sensors measuring pressure at the air delivery line (PT417) and inside the three-phase splitter (PT501). Other sensors are located at the water delivery line (FIC101, FT102), at the bottom of the two-phase splitter(FT406), at the top of the two-phase splitter (FT404), at the top of the three-phase splitter (PIC501), at the air delivery line (FIC302, FT302), at the top of the water tank (LI101), at the bottom of the three-phase splitter (LI502), and at the top of the water coalescer (LI503).

### 4.2. Fault Injection and Distributed Fault Detection

**Figure 5:**
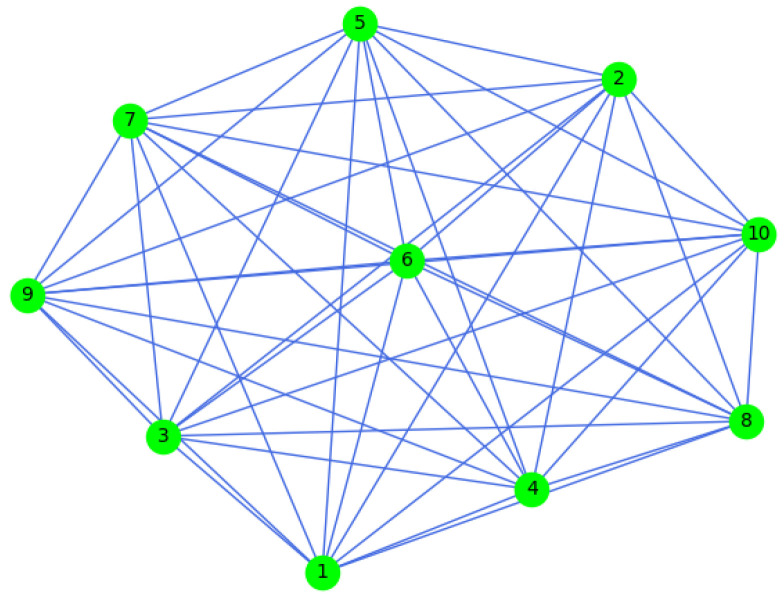
A connectivity graph of the sensor network.

In order to gather the necessary historical data for this experiment, a SCADA platform can be utilized at a sample rate of 1 Hz. The data parameters utilized in this validation are outlined in Table 1. A communication topology of the sensor network is represented in Figure 5.

**Figure 6:**
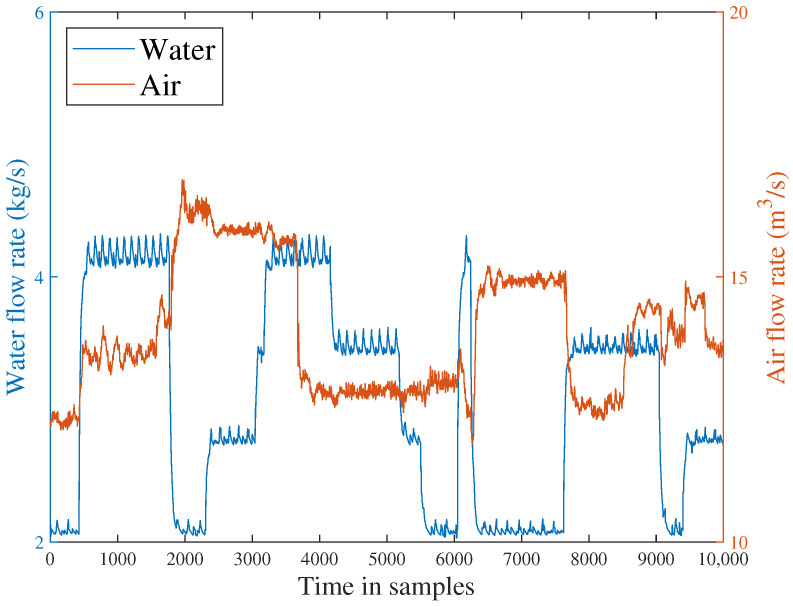
Input stream sequences for fault-free data set.

**Figure 7:**
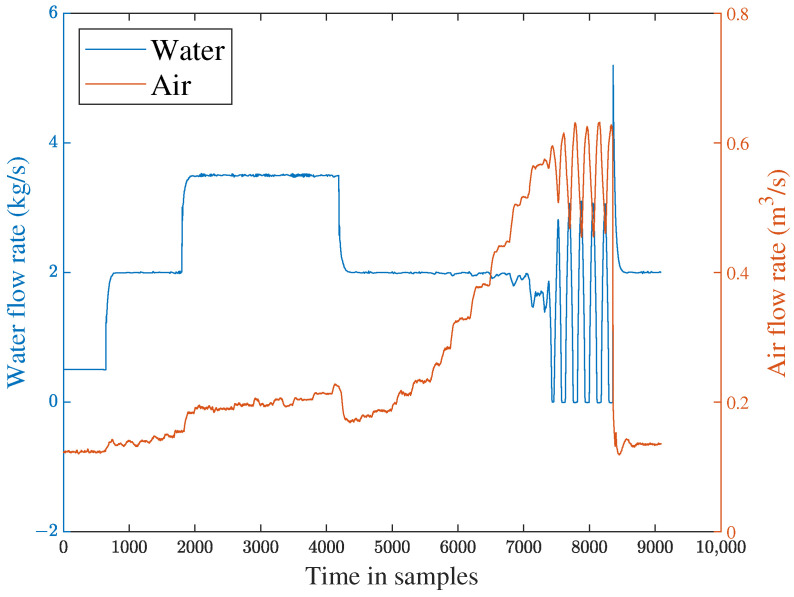
Input stream sequences for fault scenario 1.

**Figure 8:**
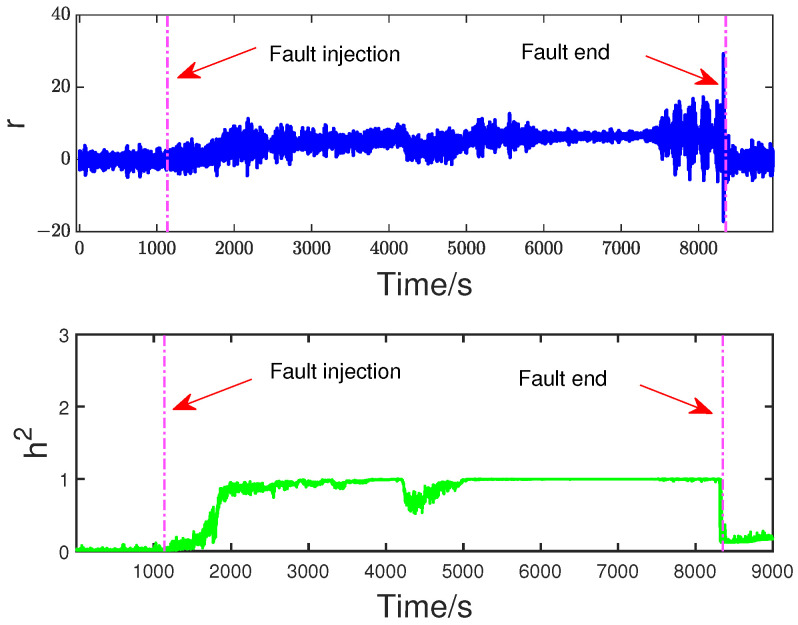
Residual signal *r* and $h^{2}$ at node 1.

To evaluate the effectiveness of the proposed method, the off-line part is first executed. The input flow rate of the training dataset is shown in Figure 6. Then, two typical faults are used to verify FD performance. The first fault scenario involves an incipient fault that arises due to the obstruction of the top separator input, leading to the shutdown of VC404 between 1136 s and 8352 s. The input situation of the faulty dataset is depicted in Figure 7. The residual signal of fault 1 and the HD-based $h^{2}$ statistic at node 1 are shown in Figure 8. The residual signal $r_{1}\left(k\right)$ in Figure 8 indicates the overall trend of the system. However, when the fault amplitude is low, the residual signal is often not effective in capturing abnormal cases. The blue curve in Figure 8 does not changed significantly after the fault occurred.

**Figure 9:**
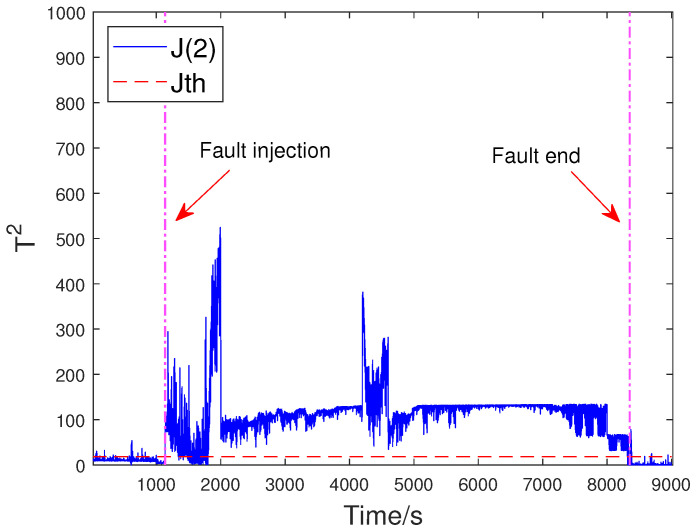
Detection results for fault 1 at node 2.

**Figure 10:**
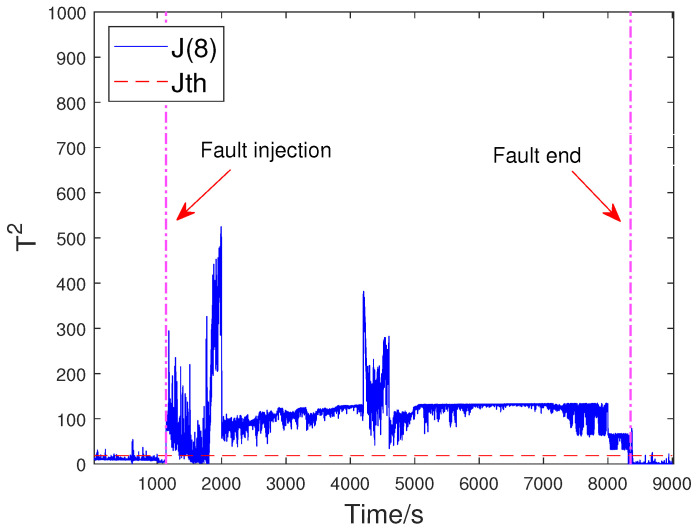
Detection results for fault 1 at node 8.

**Figure 11:**
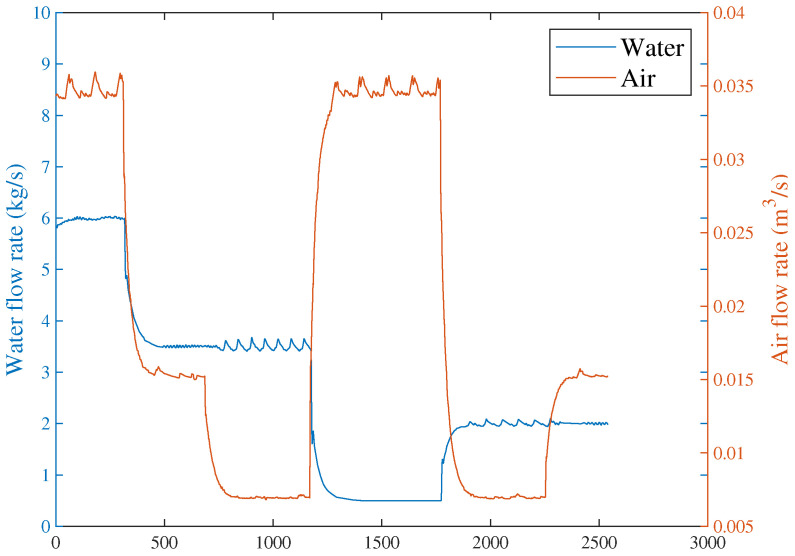
Input stream sequences for fault 2.

**Figure 12:**
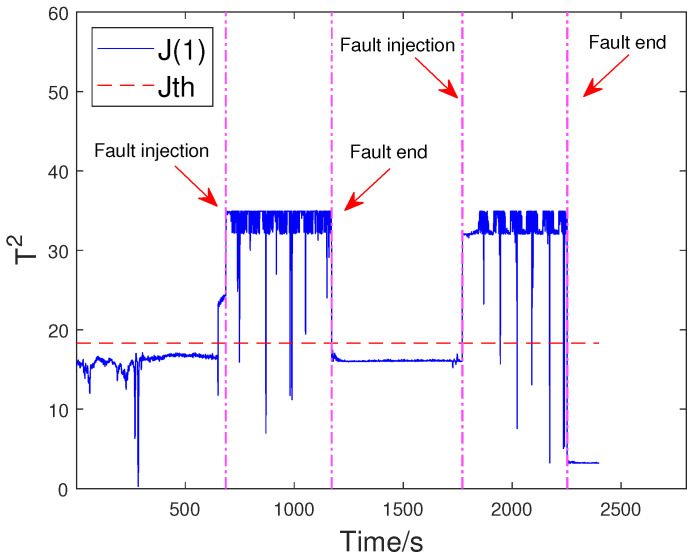
Detection results for fault 2 at node 1.

**Figure 13:**
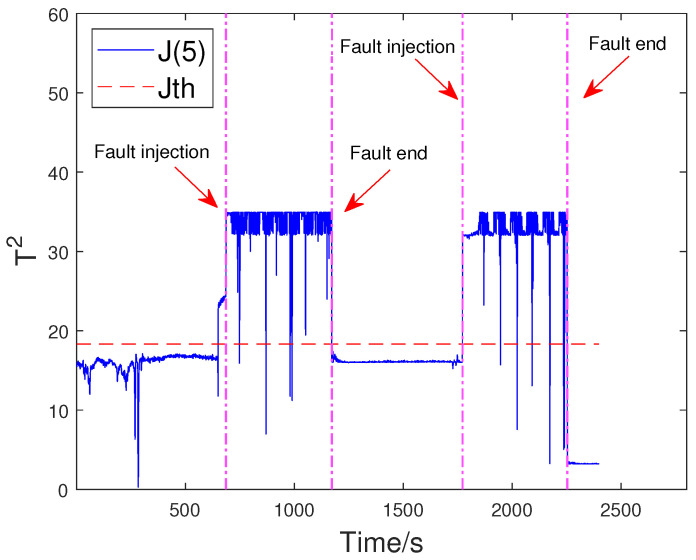
Detection results for fault 2 at node 5.

Based on the above problem, a HD-based metric is implemented into the proposed distributed SKR framework to enhance the sensitivity of the residual signal. When the fault intensity is minor, the information on the probability distribution also changes remarkably. As a result, the sensitivity of FD is significantly increased by measuring the HD of the residual signal. The green curve in Figure 8 indicates that the HD-based $h^{2}$ statistics have changed significantly after the fault occurred. The performance evaluation focuses on two key metrics: the Missed Alarm Rate (MAR) and the False Alarm Rate (FAR). In this study, the consensus algorithm is embedded in the information interaction among sensor blocks. Each node in the sensor network is used to execute FD algorithm through average consensus techniques. As a consensus result, each sensor node can obtain an identical FD performance. The distributed FD diagrams for fault 1 are displayed in Figure 9 and Figure 10. J(2) and J(8) in Figure 9 and Figure 10 represent the distributed FD results at node 2 and node 8, respectively. In terms of performance metrics, the MAR is 0.0323 and the FAR is 0.0391 at node 2. In addition, the MAR is also 0.0323 and the FAR is also 0.0391 at node 8. The second fault scenario is an intermittent fault, also known as segment plugging in practical engineering terms. This type of fault commonly occurs in the riser of multiphase flow when the flow rate of liquid and gas is low. The fault was introduced by deliberately reducing the air and water flow rates to levels that induce plugging. In this dataset, two plugging conditions were introduced and eliminated, from 686 s to 1172 s and from 1772 s to 2253 s, during the experiment. Specifically, the plugging fault was first formed at 686 s by continuously reducing the flow rate of air and water. The plugging fault was then removed at 1172 s when the air flow rate gradually increases. Additionally, the plugging fault was introduced again from 1772 s to 2253 s by changing the input flow rate. The input situation and detection results for fault 2 are shown in Figure 11, Figure 12 and Figure 13, respectively. The calculated MAR and FAR at node 1 and node 5 are 0.0610 and 0.0454, respectively.

### 4.3. Comparison Results

In order to show the enhanced FD performance, Table 2 provides four sets of evaluations using MARs and FARs as assessment indicators. In Table 2, traditional SKR and dynamic principal component analysis are centralized designs; The distributed CCA and the proposed scheme are distributed designs.

According to the performance indicators in Table 2, both FAR and MAR of the proposed algorithm are significantly lower than other FD algorithms. This indicates that the accuracy and effectiveness of the proposed algorithm are better than the traditional SKR algorithm and other comparison algorithms. The excellent FD performance is mainly the result of the introduction of the Hellinger distance in the SKR framework. Specifically, the Hellinger distance is first introduced into the traditional SKR framework to further analyze the fault features of the residual signal. Since the Hellinger distance can accurately measure the difference between two probability distributions, the proposed algorithm is more sensitive to the fault information of the residual signal. As a result, the proposed algorithm can capture the fault information in the residual signal more effectively, which further improves the reliability and accuracy of the FD algorithm.

## 5. Conclusions

This study proposes a novel distributed FD method by introducing Hellinger distance and average consensus algorithm in the SKR framework. The proposed algorithm has the following three main advantages and differences over existing FD methods. This study introduces the first Hellinger distance in the traditional SKR framework to further analyze the fault features of the residual signal. Since the Hellinger distance can accurately measure the difference between two probability distributions, the proposed algorithm is more sensitive to the fault information of the residual signal. In addition, the consensus algorithm is embedded in the information interaction among sensor blocks. Based on this idea, each block can obtain FD results without performing global fusion operations. Finally, the proposed algorithm can identify noise terms and the residual signals directly from the process data. It has superior flexibility in the design of the detection framework, particularly when the system models are not accurately obtained. The accuracy and validity of the proposed FD algorithm have been verified via a multiphase flow facility. In addition, fault-tolerant control based on data-driven SKR is an open problem that can avoid the complex design of control systems. Based on this study, distributed FD with external disturbances and fault-tolerant control based on data-driven SKR will be explored in our future work.

## Figures and Tables

**Table 1:** Parameters used in this experiment.

Sensor Location	Description	Units
PIC501	Placement of PIC501 valve	(%)
PT417	Pressure in the blending area	barg
FIC302	Placement of FIC302 valve	(%)
FIC101	Placement of FIC101 valve	(%)
FT404	Air flow rate from 2-phase splitter	m${}^{3}$/h
FT406	Water flow rate from 2-phase splitter	kg/s
PT501	Pressure in 3-phase splitter	barg
LI101	Level of water tank	m
LI502	Level of 3-phase splitter	(%)
LI503	Level of watercoalescer	(%)
FT302	Air intake velocity	Sm${}^{3}$/h
FT102	Water intake velocity	kg/s

**Table 2:** Performance comparisons for the multiphase flow plant.

Detection Framework	FD Strategies	$f_{1}:$ Blockage in the Input Block Separator			$f_{2}:$ Slugging Situation		Obtain FD Result’s Way
		MAR	FAR		MAR	FAR	
Centralized	Traditional SKR [40]	0.0452	0.0729		0.5036	0.1693	Central node
	Dynamic principal component analysis [36]	0.6417	0.3461		0.5826	0.2754	Central node
Distributed	The developed HSKR	0.0323	0.0391		0.0610	0.0454	Any node
	Distributed CCA [29]	0.3881	0.2668		0.2076	0.3045	Any node

## Data Availability

The data generated by this work are available upon reasonable request to the corresponding author.
